# Connections between Parental Emotion Socialization and Internalizing Problems in Adolescents: Examining the Mediating Role of Emotion Regulation Strategies and Moderating Effect of Gender

**DOI:** 10.3390/bs14080660

**Published:** 2024-08-01

**Authors:** Xiaowei Guo, Ruichao Jiao, Jingxin Wang

**Affiliations:** 1 Faculty of Psychology, Tianjin Normal University, Tianjin 300000, China; 1901030042@stu.tjnu.edu.cn (X.G.); jrc6767@hueb.edu.cn (R.J.); 2 School of Educational Sciences, Xinxiang University, Xinxiang 453000, China; 3 Student Affairs Office, Hebei University of Economics and Business, Shijiazhuang 050000, China

**Keywords:** parental emotion socialization, internalizing problems, adolescents, cognitive reappraisal, expressive suppression

## Abstract

To explore the mediating effect of emotion regulation strategies on the correlation between parental emotion socialization and internalizing problems in adolescents, as well as the moderating effect of gender, a questionnaire survey was administered to 1078 junior high school students (M_age_ = 13.96 ± 1.00). The results revealed that supportive parental emotion socialization was negatively correlated with adolescents internalizing problems, whereas non-supportive parental emotion socialization was positively correlated with such problems. Cognitive reappraisal and expressive suppression strategies functioned as parallel mediators in the relationship between supportive parental emotion socialization and adolescent internalizing problems, while only expressive suppression mediated the correlation between non-supportive emotion socialization and adolescent internalizing problems. Gender did not exhibit a moderating effect on the mediation model. These findings suggest that supportive parental responses to adolescents’ negative emotions can reduce the incidence of depression and anxiety by cultivating increased utilization of cognitive reappraisal strategies and decreased reliance on expressive suppression strategies among adolescents, whereas non-supportive responses exacerbate the occurrence of depression and anxiety by promoting greater utilization of expressive suppression strategies. In addition, no significant gender differences were observed in the mediation effects. These findings emphasize the importance of prevention programs focusing on parental emotion socialization in adolescence.

## 1. Introduction

Adolescent mental health work constitutes a critical component in the development of a Healthy China. Adolescence is characterized by continuous social, cognitive, and neurobiological development [1], presenting a myriad of challenges. As adolescents navigate increasingly complex developmental tasks, they may encounter difficulties and exhibit problematic behaviors, including both externalizing behaviors, such as withdrawal, aggression, and rule breaking, and internalizing behaviors, such as depression and anxiety. Internalizing problems pertain to issues in an individual’s psyche that are less readily observable and identifiable compared to externalizing problems, posing no direct threat to others [2]. In addition, the developmental trajectories of internalizing and externalizing problems differ [3], with the incidence of internalizing problems, notably depression, exhibiting a marked increase during the transition to adolescence, reaching its peak in mid-adolescence [4]. The high prevalence of internalizing problems during adolescence can precipitate negative events (including non-suicidal self-injury or even suicide), thereby significantly affecting the healthy development of adolescents [5,6,7]. Factors influencing the development of internalizing problems in adolescents have long been a central focus of research. Researchers typically approach this issue through the study of individual, environmental, and interactive factors [8], aiming to explain the underlying causes and propose potential intervention strategies.

The psychological adaptation of children and adolescents is often influenced by the family environment. As the primary agents of emotion socialization, parents significantly affect their children’s emotional development [9]. The emotional reactions of individuals are shaped by their parents’ responses to emotions, how they discuss feelings, and even their own expressions of emotion. This process, known as parental emotion socialization, is a powerful tool for regulating emotions and optimizing social interactions effectively [10,11]. Several empirical studies have shown a relationship between parental emotion socialization and mental health outcomes [3,12,13,14,15,16,17,18,19,20,21,22,23]. Nonetheless, there are discrepancies in the results regarding adolescent populations [3,24]. Further examination of the processes by which parental emotion socialization impact the emotional development of adolescents and the consequent impact on the development of internalizing problems is necessary to enhance our strategies of prevention and intervention for these problems.

### 1.1. Parental Emotion Socialization and Adolescent Internalizing Problems

Socialization begins with parent–child interactions, helping the individual to perceive, identify, and regulate emotions [24]. According to the theory of emotion socialization, parental emotion socialization has the capability to mold children’s ability of emotion understanding, expression, and regulation through modeling, emotional climate, and emotion socialization practices [10,11]. While modeling and emotional climate exert their influence indirectly, emotion socialization practices represent a more direct approach [10,25]. When parents respond to their adolescent children’s emotional expressions, they are engaging in emotion socialization practices. These practices involve deliberate behaviors aimed at providing immediate and specific feedback. This feedback focuses on the acceptability of the displayed emotion, its potential causes, and effective strategies for regulation.

Parental reactions to negative emotional displays in children are commonly categorized as either supportive or non-supportive [9]. Supportive parental responses include a range of behaviors, including offering comfort and encouragement, validating a child’s feelings, and engaging in open discussions about the origins and potential consequences of their emotions. Research suggests that parents who had more supportive responses to their children’s emotions (e.g., comforting, encouraging, and acceptance) were more likely to establish stable and secure parent–child relationships, exhibit more adaptive behaviors, and display fewer behavioral problems [26]. Conversely, non-supportive responses, often characterized by parental dismissal or punishment of negative emotional expression, communicate to children that such emotions are unacceptable and should be suppressed. This can lead adolescents to develop patterns of emotional avoidance, hindering their ability to accurately recognize, process, and express their emotions, leading to worse maladaptive and problematic behavior [12,27]. Neuroscientific research provides further evidence for the impact of parental responses to children’s emotions on the development of emotional regulation and associated brain function. For instance, Tan et al. found that non-supportive maternal responses can inhibit the activity of neural networks associated with processing social rewards during peer interactions in adolescence [28]. Conversely, utilizing fMRI technology, Lee et al. demonstrated that supportive maternal responses can modulate the activation of emotional networks, including the subcortical limbic region, in adolescents when faced with criticism [29]. This finding suggests that in a warm and supportive parent–child relationship, criticism may carry less potential for negative impact. A meta-analytic study has indicated a significant correlation between parental emotion socialization and both immediate and prospective problem behaviors in individuals [25].

There have been studies on the relationship between parental emotion socialization and internalizing problems with a greater focus on children. During adolescence, a period of significant emotional growth, youth experience a decline in reliance on parents along with a growth in the role of peers, romantic interests, and other social connections. Although some scholars argue that the impact of parental emotion socialization diminishes as adolescents get older, with other socialization agents at the fore [3], others maintain that parental emotion socialization is key to nurturing emotional development throughout adolescence [11,24]. Adolescents are heavily dependent on parental support, in particular for their emotional regulation, particularly for negative events and emotions [30]. Despite the extensive research conducted, further examination will be able to expose the thorough mechanisms through which parental emotion socialization influences adolescent growth. 

Built on diverse parental perspectives on emotion expression and regulation, and situated in varying family and cultural influences, achieving a consensus on a positive model of parental emotion socialization remains challenging [10]. Chinese families, however, display unique patterns and features of emotion socialization that differ from the Western framework. Consequently, parental reactions to childhood emotional expressions may communicate different signals and carry different meanings when they occur in this cultural context [31]. A prime example is that Chinese families highly value the nurturance of relational emotional competencies and promote emotional regulation or concealment that focuses on personal needs [32,33]. This cultural emphasis on emotional control might cause a less detrimental consequence associated with low emotional expression. However, the studies that exist mainly concentrate on Western family models which stresses the demand to assess the consequence of parental emotion socialization on child performance in diverse cultural backgrounds, especially non-Western cultures [34]. Based on this, a deeper understanding of how Chinese parents’ emotion socialization practices affect adolescents in internalizing problems can offer culturally sensitive and suitable guidance for Chinese families.

### 1.2. Mediating Role of Emotion Regulation

Emotion regulation comprises the processes by which individuals modulate their responses to psychological and environmental stimuli, involving the intentional or automatic deployment of strategies aimed at changing cognitive and behavioral patterns, finally influencing the generation, experience, and expression of emotions in pursuit of individual goals [35,36,37,38,39]. 

Difficulties in emotion regulation are widely recognized as a transdiagnostic factor implicated in a range of adolescent psychopathology [37,40,41,42]. Two meta-analyses have demonstrated a moderate correlation between adolescents’ habitual use of emotion regulation strategies and the presentation of internalizing symptoms [35,43]. The Extended Process Model (EPM) of emotion regulation identifies cognitive reappraisal and expressive suppression as key factors correlated with internalizing behaviors in adolescents [38]. As two extensively studied emotion regulation strategies, reappraisal effectively reduces the experiential and behavioral components of negative emotions in situations requiring their downregulation. Habitual reappraisers tend to report fewer negative emotions, suggesting reappraisal may protect against vulnerability to depression and anxiety. While expressive suppression is generally considered maladaptive in Western cultures, this view is not universally shared, as collectivistic cultures, such as China, do not necessarily perceive it as such [44]. A growing body of evidence suggests a significant correlation between emotion regulation and the development and persistence of psychopathology in adolescents [37,40,42,45,46]. This relationship appears to be more significant in internalizing symptoms compared to externalizing symptoms [42]. In effect, emotion regulation is recognized as a critical factor in the developmental trajectory of internalizing problems in adolescents [41]. Intervention studies also support that children’s use of maladaptive emotion regulation strategies predicts emotional problems [47].

Adolescence is a critical period for the development of emotion regulation. Emotion regulation is driven jointly by one’s tendency to choose a specific regulatory strategy and one’s capacity to implement said strategy effectively [39]. Parental emotion socialization influences both the tendency and capacity with which adolescents utilize emotion regulation strategies. Numerous studies have explored the impact of parental emotion socialization on the development of emotion regulation and social adaptation in adolescents, focusing primarily on the performance of emotion regulation abilities [12,13,20,21,48,49,50]. However, there is a significant gap in research exploring the impact of utilizing specific emotion regulation strategies [14,15,18,27]. While Herd et al. found that the use of emotion regulation strategies, particularly cognitive reappraisal, was less susceptible to parental influence [51,52], further research is needed in this area. Moreover, the effects of supportive and non-supportive responses of parental emotion socialization appear to differ between childhood and adolescence. Castro et al. suggested that when developmentally the positive effects of parental supportive responses diminish, non-supportive responses become less harmful or even helpful to facilitate the socioemotional competence development [53]. From this perspective, supportive responses might impede adolescents’ autonomous regulation development whereas non-supportive responses by indicating high parental expectations for self-regulation may inadvertently enhance its effectiveness [54]. Therefore, the use of emotion regulation strategies may play a mediating role in the relationship between parental emotion socialization and adolescents’ internalization problems.

### 1.3. Moderation Role of Gender

Children’s gender can affect how parents respond to their negative emotions [10]. For instance, parents are more likely to respond supportively to girls’ negative emotions and are more prone to respond in minimizing and controlling ways to boys experiencing sadness and fear [9]. In addition, girls and boys have different expectations regarding how their parents will respond to their negative emotions.

The process of emotion socialization intertwines with gender socialization, influencing how children regulate emotions and adapt socially. While research confirms the influence of parental emotion socialization on children of different genders, specific findings require further clarification. For instance, Song et al. indicated a correlation between non-supportive maternal responses and increased negative emotions exclusively in girls [55]. Similarly, Gaté et al. determined that rumination mediated the relationship between less positive maternal parenting and internalizing symptoms only in the female participant group [27]. The results of another longitudinal study also indicated that rumination mediates the pathway from mothers’ severe discipline to girls’ anxiety and depression symptoms, while controlling for baseline anxiety and depression symptoms; however, the mediating path was not significant for boys [14]. While some studies indicate that boys may be more susceptible to the effects of parental socialization on depression [56], others present contrasting findings. For instance, Perry et al. found no significant gender differences in the relationship between mothers’ supportive and non-supportive responses to negative emotions in five-year-old children and later adjustment problems at age fifteen [57]. This suggests that gender alone may not be the sole determining factor. Some researchers, such as Liu et al., emphasized the interaction between gender and temperament in understanding how parental emotion socialization influences children [58]. Further research is crucial to unravel the moderating role of gender in the relationship between parental emotion socialization and adolescents’ emotional development and social adjustment.

### 1.4. Current Study

Considering the inconsistencies between adolescents’ perceptions of parental caregiving and caregivers’ self-reports [59,60,61,62,63], as well as evidence indicating that children’s perceptions of parental caregiving have a stronger impact on children’s emotions and well-being than do parental reports [59,63,64], this study focuses on adolescents aged 12–15 years old, utilizing adolescents’ self-reported parental responses to their negative emotions as indicators of parental emotion socialization, with emotion regulation strategies as the mediating variable and gender as the moderating variable. The study aims to explore the effect of parental emotion socialization on adolescent depression and anxiety, aiming to further enrich research in this field and offer evidence for interventions in adolescent emotion regulation and mental health education.

**Figure 1:**
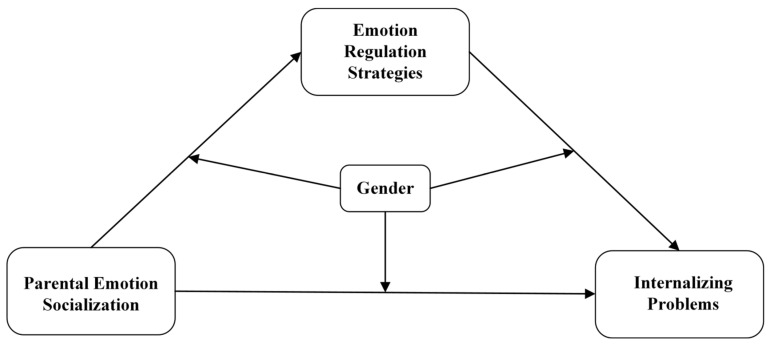
Hypothetical Model of the Study.

Building upon the existing literature, we hypothesize that parental responses to adolescent expressions of negative emotions may affect the occurrence of internalizing problem symptoms in adolescents through their effect on adolescents’ adoption of specific emotion regulation strategies. The initial hypotheses guiding this analysis are outlined below (Figure 1).

**Hypothesis 1.**  *Supportive parental emotion socialization negatively predicts adolescent internalizing problems, while non-supportive parental emotion socialization positively predicts adolescent internalizing problems.*

**Hypothesis 2.**  *Supportive parental emotion socialization decreases internalizing problems by increasing the use of cognitive reappraisal strategies and decreasing the use of expressive suppression strategies, whereas non-supportive parental emotion socialization increases internalizing problems by reducing the use of cognitive reappraisal strategies and increasing the use of expressive suppression strategies.*

**Hypothesis 3.**  *Adolescent gender moderates the pathway from parental emotion socialization to emotion regulation strategies to internalizing problems.*

## 2. Methods

### 2.1. Participants

A stratified sampling methodology was employed to select four junior high schools situated in Xinxiang City, Henan Province. The sample comprised two schools located in urban areas, one situated in the county seat, and one located in a township. Two classes were randomly selected from each grade level in the chosen schools. Trained psychology students administered questionnaires collectively in the selected classrooms. Of the 1200 distributed questionnaires, 1161 were returned. Based on the exclusion criteria, such as regularity of filling in and consecutive omission, 83 questionnaires were excluded and 1078 valid responses were retained, with an effective response rate of 92.9%. The demographic characteristics of the respondent sample were as follows: 545 male respondents and 533 female respondents, with an average age of 13.96 ± 1.00 years (age range: 12–16). The sample included 129 children who were the only child in the family and 948 children who were not the only child in the family. Grade level distribution was as follows: 408 seventh graders, 327 eighth graders, and 343 ninth graders. Parental education levels were reported as follows: 33 fathers had attained primary education or below, 402 had completed junior high school, 291 had completed high school or technical secondary school, 151 had attended college (including junior college), and 50 had obtained a master’s degree or higher. For mothers, 32 had attained primary education or below, 438 had completed junior high school, 265 had completed high school or technical secondary school, 135 had attended college (including junior college), and 59 had obtained a master’s degree or higher. Parental education level information was not offered by 149 respondents.

### 2.2. Measures

#### 2.2.1. Parental Emotion Socialization

Parental emotion socialization was assessed using the Emotions as a Child Scale (EAC; child version), which was initially developed by Magai and O’Neal in 1997 [65] and revised by Luo et al. in 2020 [66]. This scale evaluates parental emotion socialization strategies concerning three negative emotions in children: anger, fear, and sadness. It constitutes a revised scale of 36 items, with 12 items for each anger subscale (e.g., “When I was angry, my parents told me to stop being angry”), fear subscale (e.g., “When I was afraid, my parents helped me deal with the issue that made me fear”), and sadness subscale (e.g., “When I was sad, my parents got very sad.”), respectively. Each subscale is made up of two indicators, namely supportive responses and non-supportive responses. The participant is asked to rate how typical each response is on a five-point Likert scale (1 = “never”, 5 = “very often”). In this study, the Cronbach’s α coefficient for the overall scale was 0.89, while the coefficients for the subscales were as follows: anger subscale (0.63); fear subscale (0.75); sadness subscale (0.78).

#### 2.2.2. Emotion Regulation Strategies

The Emotion Regulation Questionnaire (ERQ) formulated by Gross and John [67] and revised by Wang et al. [68] was adopted in this study. The scale includes 10 items and is divided into cognitive reappraisal (e.g., “I control my emotions by changing the way I think about the situation I’m in”) and expressive suppression (e.g., “I control my emotions by not expressing them”) subscales. All items are rated on a seven-point Likert scale (1 = “strongly disagree”, 7 = “strongly agree”). Higher scores above the midpoint mean that a higher frequency was used for the given strategy. The Chinese version of the ERQ possessed sound psychometric properties. The Cronbach’s α coefficient for cognitive reappraisal subscale was 0.80, and the Cronbach’s α coefficient for expressive suppression subscale was 0.81 in the present study.

#### 2.2.3. Adolescent Internalizing Problems

Adolescent internalizing problems were assessed as anxiety and depressive symptoms. In order to measure adolescent depressive symptoms, we administered the Center for Epidemiologic Studies Depression Scale (CESD) [69], which is a 20-item self-report measure of depression. The CESD focuses on depressive affect and mood, enabling comparisons of survey data across different time points to track changes in depressive symptom scores. As such, it is particularly well-suited for assessing depressive symptoms in non-clinical populations. Participants were instructed to reflect over the past week and indicate the extent to which they experienced each item on a four-point Likert scale (0 = “rarely or none of the time”; 1 = “some of the time”, 2 = “occasionally or moderate amount of time”, 3 = “most or all of the time”). Four positive items were reverse-scored, and items were summed to obtain total scores; higher scores indicated greater levels of depression. This measure has been used extensively in previous research on adolescents. In this study, the Cronbach’s α coefficient for the CESD was 0.83.

This study employed the State-Trait Anxiety Inventory, developed by Spielberger et al., to evaluate anxiety levels among adolescents. The State Anxiety Inventory comprises 20 items, with half addressing negative emotions and the other half focusing on positive emotions. It analyzes an individual’s current or situation-specific experiences of fear, tension, worry, and nervousness. A four-point Likert scale is used, ranging from 1 being “not at all”, 2 being “somewhat”, 3 being “moderately”, to 4 being “very much”. Ten items are reverse-scored, resulting in higher total scores reflecting greater anxiety levels. The Cronbach’s α coefficient for the State Anxiety Inventory in this study was 0.89.

### 2.3. Procedure and Data Analysis

This study was approved by the Ethics Review Board of the author’s university and was conducted by a group of students trained in psychology. Informed consent was obtained from school leaders, class teachers, students, and parents. Participating students received a stationery gift.

The raw data were screened to eliminate questionnaires with systematic or excessive missing responses, resulting in the final dataset. Descriptive statistical analysis and correlation analysis were performed for all variables utilizing SPSS 26.0. Mplus 8.0 was utilized to test the hypothesized model, specifically analyzing the mediating effect of adolescent emotion regulation strategy use on the correlation between parental emotion socialization and depression/anxiety, as well as the moderating effect of gender on these pathways. Bootstrap resampling with 5000 repetitions was employed to generate 95% confidence intervals for the specified paths. Model fit was assessed based on the chi-square statistic (χ^2^), Comparative Fit Index (CFI), Root Mean Square Error of Approximation (RMSEA), and Standardized Root Mean Square Residual (SRMR). A model fit was considered acceptable if CFI > 0.90, RMSEA < 0.08, and SRMR < 0.08 [70].

## 3. Results

### 3.1. Common Method Bias Test

Recognizing the limitations of self-report data, specifically the potential for artificial covariation between independent and dependent variables originating from a single data source [71], the study implemented measures to reduce common method bias. The Harman single-factor test was employed to assess the extent of variance attributable to a single latent factor. The results indicate that the first factor accounted for 22.57% of the total variance, less than the critical value of 40%. Therefore, it is considered that no serious common method bias is present.

### 3.2. Descriptive Statistics and Correlation Analysis

Adolescent internalizing problems, as measured by depression and anxiety scores, are presented in Table 1. Gender variables were treated with dummy coding and incorporated into the correlation analysis. Depression and anxiety scores demonstrate significant gender differences. Adolescent girls exhibit significantly higher levels of both depression and anxiety compared to boys (depression: 19.49 ± 11.77 vs. 17.68 ± 10.78; r = 0.08, *p* = 0.01; anxiety: 43.72 ± 10.57 vs. 41.24 ± 10.30; r = 0.12, *p* < 0.001). No significant correlation was observed between age and the scores of any variables. Supportive parental emotion socialization exhibited a positive correlation with the utilization of cognitive reappraisal strategies (r = 0.38, *p* < 0.001) and a negative correlation with expressive suppression (r = −0.26, *p* < 0.001). In addition, supportive parental emotion socialization negatively correlated with both anxiety (r = −0.40, *p* < 0.001) and depression (r = −0.39, *p* < 0.001), whereas non-supportive parental emotion socialization was negatively correlated with cognitive reappraisal strategy usage (r = −0.12, *p* < 0.001) and positively correlated with expressive suppression (r = 0.26, *p* < 0.001). Moreover, non-supportive parental emotion socialization demonstrated positive correlations with anxiety (r = 0.31, *p* < 0.001) and depression (r = 0.36, *p* < 0.001).

### 3.3. Mediation Effects Analysis of Emotion Regulation Strategies

**Figure 2:**
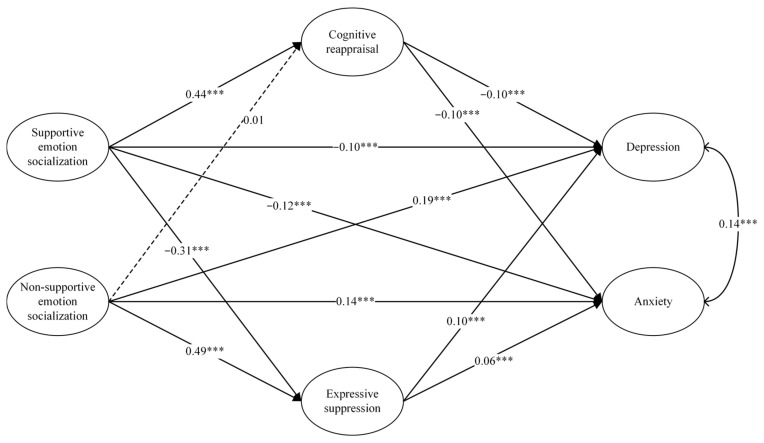
Mediating model of emotion regulation strategies between parental emotion socialization and Internalizing Problems. Note: The solid lines represent the significant paths and the dashed lines represent the non-significant ones. All paths are represented by standardized coefficients. *** *p* < 0.001.

The parallel mediation pathway model included supportive and non-supportive emotion socialization as independent variables, depression and anxiety scores as dependent variables, and cognitive reappraisal and expression suppression as mediating variables, controlling for gender. The model demonstrated a good fit: χ^2^ (1) = 8.20, *p* < 0.001, CFI = 0.99, TLI = 0.92, RMSEA = 0.08, SRMR = 0.02. All mediation effects were significant except for the mediation effect of cognitive reappraisal on the relationship between non-supportive parental emotion socialization and depression/anxiety (Figure 2).

Table 2 and Table 3, which present the results of the study using adolescent depressive symptoms as dependent variables, demonstrate a direct correlation between supportive emotion socialization and depression (β = −0.10, 95%CI = [−0.14, −0.06]). This relationship is partially mediated by both cognitive reappraisal (β = −0.04, 95%CI = [−0.06, −0.03]) and expressive suppression (β = −0.03, 95%CI = [−0.05, −0.02]). It is important to note that the difference between the mediating effects of cognitive reappraisal and expressive suppression is not significant. In addition, non-supportive emotion socialization exhibits a direct relationship with depression (β = 0.19, 95%CI = [0.14, 0.25]). Expressive suppression partially mediates this relationship (β = 0.05, 95%CI = [0.03, 0.07]), while the mediating effect of cognitive reappraisal is not significant. This model successfully accounts for 32.1% of the variance observed in adolescent depressive emotions.

Table 2 and Table 3 indicate the results of the study using adolescent anxiety symptoms as dependent variables. As presented in Table 2, the research findings indicate a direct correlation between supportive emotion socialization and anxiety (β = −0.12, 95%CI = [−0.15, −0.08]). Both cognitive reappraisal (β = −0.04, 95%CI = [−0.06, −0.03]) and expressive suppression (β = −0.02, 95%CI = [−0.03, −0.01]) partially mediate this relationship. Specifically, the mediating effect of expressive suppression is significantly stronger than that of cognitive reappraisal. Non-supportive emotion socialization also demonstrates a direct relationship with anxiety (β = 0.14, 95%CI = [0.10, 0.19]), with expressive suppression partially mediating this relationship (β = 0.03, 95%CI = [0.02, 0.05]). However, the mediating effect of cognitive reappraisal is not significant. This model effectively explains 27.9% of the variance observed in adolescent anxiety.

### 3.4. Moderation Effects Analysis of Gender

**Figure 3:**
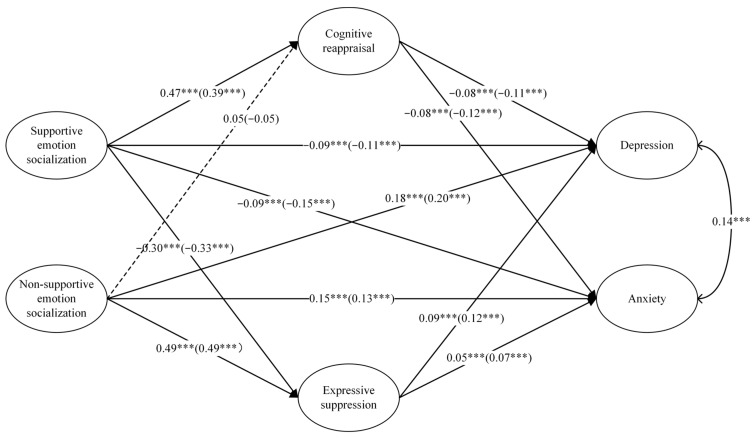
Mediating model of gender as moderating variable. Note: Path coefficients outside parentheses for males, inside parentheses for females. The solid lines represent the significant paths and the dashed lines represent the non-significant ones. All paths are represented by standardized coefficients. *** *p* < 0.001.

The moderating role of gender was analyzed through multi-group analysis, specifically by conducting an analysis of the mediation model on the gender variable. This analysis aimed to test for gender differences in the path coefficients of the models for boys and girls. Significant differences between the models would indicate a moderating effect of gender. The results, as indicated by the Wald test results (*p*s > 0.05), indicated no significant differences in the mediation model based on the gender variable. A detailed visual representation of these findings can be found in Figure 3.

## 4. Discussion

### 4.1. The Influence of Parental Emotion Socialization on Adolescent Emotion Regulation Strategy Use and Internalizing Problems

Following the COVID-19 pandemic, a significant increase in the internalization of psychological distress has been observed among adolescents [72]. This study indicated that parental emotion socialization influences the feelings of depression and anxiety in adolescents. Non-supportive parental responses can lead adolescents to conceal and accumulate negative emotions, contributing to internalization issues. Conversely, supportive responses can facilitate the expression and acknowledgment of negative emotions in adolescents, aiding them in coping and adapting, thereby reducing the build-up of negative emotions and the associated internalization. These findings largely align with previous studies [10,23,73]. However, when analyzing the impact on internalizing problems in Chinese adolescents with more specific parental emotion socialization strategies as independent variables, the results were inconsistent [58,73]. It is worth noting that the Chinese version of the EAC in this study is based on a revised two-factor model of parental emotion socialization [66], and the results were in line with previous attempts to integrate inhibitive strategies (e.g., “Told me to stop being angry/fear/sadness”) into supportive responses. Therefore, it is crucial to differentiate the specific reactions of supportive and non-supportive parents in diverse cultures.

Emotion socialization is a process that extends beyond the direct transfer of skills from parents to children [10]. The child’s own capacity for emotion regulation is a crucial mediating factor. The results indicate that the significant comorbidity between depression and anxiety establishes a strong correlation between these conditions, enabling them to function collectively as prominent indicators of internalizing problems. Therefore, similar pathways of influence emerge concerning parental emotion socialization’s effect on adolescent depression and anxiety. In addition, in supportive parental emotion socialization, both the reduction in expressive suppression and the enhancement of cognitive reappraisal can contribute to a reduction in depression and anxiety. Specifically, regarding their effect on depression, both strategies exhibit similar mediating effects. However, concerning anxiety, expressive suppression demonstrates a more significant mediating effect, whereas it is through expressive suppression (as opposed to cognitive reappraisal) that non-supportive emotion socialization affects depression and anxiety.

Adolescents tend to intensify their efforts to employ adaptive emotion regulation strategies when they perceive supportive parental responses to their negative emotional experiences. Supportive parental responses may, to a certain extent, reduce the intensity of negative emotional arousal. When adolescents encounter emotions of moderate to low intensity, they demonstrate a higher likelihood to utilize cognitive reappraisal as a coping mechanism [74], whereas if individuals perceive parental responses to their negative emotions as more punitive or non-supportive, they are more prone to suppress and inhibit their emotional expression. While such inhibition may prove adaptive in the immediate context, its inhibition incurs significant physiological and emotional costs [44,45]. Moreover, this suppression can lead to the encoding of these negative emotions in memory, rendering them susceptible to reactivation in similar situations, finally contributing to increased anxiety and depressive symptomatology.

Prior research indicates a correlation between supportive parental emotion socialization and effective emotion regulation, while non-supportive responses have been correlated with emotional dysregulation and problematic behaviors. However, the effect of non-supportive responses on children’s emotional functioning exhibits inconsistency. Specifically, in the Chinese cultural context, supportive responses in parental emotion socialization act as protective factors for adolescent emotion regulation strategy utilization and the internalization of problems, whereas non-supportive responses do not necessarily impede the use of cognitive reappraisal strategies and may potentially contribute to the development of depression and anxiety. This observation aligns with findings from studies conducted on Asian samples, where non-supportive responses (e.g., minimization) demonstrate no significant correlation with children’s emotion regulation and may even exhibit positive effects [32]. In the future, further research is necessary to gain a deeper understanding of the non-supportive responses that affect adolescents’ emotion regulation and adaptive behavior.

### 4.2. Gender Moderation Analysis

Some researchers have suggested that parents respond similarly to grief, anger, and fear in both sons and daughters, and that parents’ responses to emotions differ only by adolescent age and problem status [9,31]; other researchers have suggested that parental responses to negative emotions in adolescents are influenced by gender [14,27,55]. While there were significant gender differences in depression, anxiety, and cognitive reappraisal use in this study, gender did not play a moderating role in the model. This phenomenon could be attributed to the fact that the choice of an emotion regulation strategy as an indicator carries different implications and influencing factors for its adoption, as opposed to the emotion regulation effectiveness [75,76]. Conversely, it may be due to the interaction between gender influence and other variables, such as the number of offspring [77] and the temperament of offspring [58]. It is thus necessary to further explore the moderating effect of gender.

## 5. Limitations and Prospects

In this study, adolescents were selected as subjects to test the model of the influence of parental emotion socialization on emotion regulation and social adaptation [10,11,24,78]. First, the cross-sectional design employed in this study presents limitations in establishing definitive causal relationships among parental emotion socialization, emotion regulation, and adolescent internalizing problems. Future research should prioritize longitudinal tracking studies or intervention experiments to offer more robust evidence for causal inference. In addition, the study’s reliance on self-reported measures from adolescents necessitates acknowledgment of the subjectivity. Future research should strive to incorporate parental perspectives or employ multi-informant approaches to comprehensively evaluate adolescent parental emotion socialization. Secondly, this study explored the impact of parental emotion socialization on adolescent behavior from a variable-centered perspective. However, parents rarely rely on a single socialization strategy, and cultural variations in these strategies are common. Future research could benefit from a person-centered approach to better understand how diverse emotional socialization patterns influence adolescent emotional regulation and internalization problems. Third, this study focused on parental socialization responses to adolescents’ negative emotions, merging paternal and maternal emotion socialization for analysis. However, a growing body of research indicates unique roles for fathers and mothers in children’s emotion socialization processes, with increasing attention directed towards the role of fathers [18,31,79]. In the Chinese cultural context, characterized by the “strict father, kind mother” stereotype, it is important to contrast the effects of paternal and maternal emotion socialization. This could help optimize parenting practices and inform targeted family education recommendations. Finally, this study focused solely on emotion socialization practices, one crucial aspect of parental emotion socialization. Future research could expand our understanding of how the modeling of parental, along with the construction of a supportive emotional atmosphere, impact the emotional development of adolescents.

## 6. Conclusions

In summary, it is well-established that supportive parental emotion socialization can reduce both depression and anxiety in adolescents by promoting the utilization of cognitive reappraisal as an emotion regulation strategy, while simultaneously discouraging the use of expressive suppression. Both strategies have similar mediating effects on depressive symptoms. However, expressive suppression has a more significant mediating role in anxiety, and non-supportive emotion socialization affects both depression and anxiety primarily through expressive suppression. Moreover, no significant gender differences were observed in the mediation effects. These findings emphasize the importance of strengthening guidance on supportive parental emotion socialization in family education programs. Such efforts can empower adolescents to engage in adaptive emotion regulation strategies, minimize the reliance on maladaptive strategies, and therefore reduce the incidence of internalizing problems, finally cultivating healthy adolescent development.

## Figures and Tables

**Table 1:** Descriptive statistics and Correlations of All Variables.

Variables	1	2	3	4	5	6	7	8
1. Gender	1							
2. Age	−0.01	1						
3. Depression	0.08 **	−0.00	1					
4. Anxiety	0.12 ***	0.03	0.75 ***	1				
5. Supportive emotion socialization	−0.03	−0.03	−0.39 ***	−0.40 ***	1			
6. Non-supportive emotion socialization	−0.05	−0.06	0.36 ***	0.31 ***	−0.34 ***	1		
7. Cognitive reappraisal	−0.09 **	0.01	−0.29 ***	−0.32 ***	0.38 ***	−0.12 ***	1	
8. Expressive suppression	0.00	0.04	0.39 ***	0.30 ***	−0.26 ***	0.26 ***	−0.02	1
M	0.49	13.96	18.58	42.47	3.50	1.70	4.79	4.05
SD	0.50	1.00	11.31	10.50	0.98	0.62	1.13	1.58

Note: Gender is coded as 0 = boys, 1 = girls. ** *p* < 0.01, *** *p* < 0.001.

**Table 2:** The Influence of Parental Emotion Socialization on Adolescent Emotion Regulation Strategies and Internalizing Problems.

Dependent Variable	Independent Variable	R^2^	B	SE	t	95%CI
Cognitive reappraisal	Supportive emotion socialization	0.15	0.44	0.04	11.07 ***	0.36, 0.51
	Non-supportive emotion socialization		0.01	0.06	0.12	−0.11, 0.13
	Gender		−0.17	0.06	−2.63 **	−0.29, −0.04
Expressive suppression	Supportive emotion socialization	0.10	−0.31	0.05	−5.89 ***	−0.42, −0.21
	Non-supportive emotion socialization		0.49	0.08	6.24 ***	0.34, 0.64
	Gender		0.01	0.09	0.14	−0.17, 0.19
Depression	Supportive emotion socialization	0.32	−0.10	0.02	−5.18 ***	−0.14, −0.06
	Non-supportive emotion socialization		0.19	0.03	6.73 ***	0.14, 0.25
	Cognitive Reappraisal		−0.10	0.02	−6.53 ***	−0.12, −0.07
	Expressive Suppression		0.10	0.01	10.05 ***	0.08, 0.12
	Gender		0.08	0.03	2.72 **	0.02, 0.13
Anxiety	Supportive emotion socialization	0.28	−0.12	0.02	−6.33 ***	−0.15, −0.08
	Non-supportive emotion socialization		0.14	0.02	5.92 ***	0.10, 0.19
	Cognitive Reappraisal		−0.10	0.02	−6.45 ***	−0.13, −0.07
	Expressive Suppression		0.06	0.01	6.37 ***	0.05, 0.08
	Gender		0.11	0.03	3.89 ***	0.05, 0.16

Note: ** *p* < 0.01, *** *p* < 0.001. 95%CI = 95% Confidence Interval. B = Unstandardized coefficients. SE = Standard error.

**Table 3:** Mediation Effect of Emotion Regulation Strategies between Parental Emotion Socialization and Internalizing Problems.

Effect Types		Effect	Boot SE	Boot 95%CI
Direct effect	SES → depression	−**0.10**	0.02	−0.14, −0.06
	NES → depression	**0.19**	0.03	0.14, 0.25
	SES → anxiety	−**0.12**	0.02	−0.15, −0.08
	NES → anxiety	**0.14**	0.02	0.10, 0.19
Indierct effect	SES → CR → depression	−**0.04**	0.01	−0.06, −0.03
	SES → ES → depression	−**0.03**	0.01	−0.05, −0.02
	NES → CR → depression	−0.00	0.01	−0.01, 0.01
	NES → ES → depression	**0.05**	0.01	0.03, 0.07
	SES → CR → anxiety	−**0.04**	0.01	−0.06, −0.03
	SES → ES → anxiety	−**0.02**	0.01	−0.03, −0.01
	NES → CR → anxiety	−0.00	0.01	−0.01, 0.01
	NES → ES → anxiety	**0.03**	0.01	0.02, 0.05

Note: SES = Supportive Emotion Socialization; NES = Non-supportive Emotion Socialization; CR = Cognitive Reappraisal; ES = Expressive Suppression. Bolded values indicate significant effects.

## Data Availability

The dataset analyzed during the current study is not publicly available but is available from the corresponding author on reasonable request.
